# Comprehensive serial molecular profiling of an “N of 1” exceptional non-responder with metastatic prostate cancer progressing to small cell carcinoma on treatment

**DOI:** 10.1186/s13045-015-0204-7

**Published:** 2015-10-06

**Authors:** Kunal C. Kadakia, Scott A. Tomlins, Saagar K. Sanghvi, Andi K. Cani, Kei Omata, Daniel H. Hovelson, Chia-Jen Liu, Kathleen A. Cooney

**Affiliations:** Department of Internal Medicine, University of Michigan Comprehensive Cancer Center, 7216 Cancer Center, SPC 5948, 1500 East Medical Center Drive, Ann Arbor, MI 48109 USA; Department of Pathology and Urology, Michigan Center for Translational Pathology; University of Michigan Comprehensive Cancer Center, Ann Arbor, MI 48109 USA; Boonshoft School of Medicine, Wright State University, Dayton, USA; Department of Pathology, Michigan Center for Translational Pathology, Ann Arbor, MI 48109 USA

**Keywords:** Neuroendocrine prostate cancer, Small cell prostate cancer, Transdifferentiation, Next generation sequencing

## Abstract

**Importance:**

Small cell carcinoma/neuroendocrine prostate cancer (NePC) is a lethal, poorly understood prostate cancer (PCa) subtype. Controversy exists about the origin of NePC in this setting.

**Objective:**

To molecularly profile archived biopsy specimens from a case of early-onset PCa that rapidly progressed to NePC to identify drivers of the aggressive course and mechanisms of NePC origin and progression.

**Design, setting, and participants:**

A 47-year-old patient presented with metastatic prostatic adenocarcinoma (Gleason score 9). After a 6-month response to androgen deprivation therapy, the patient developed jaundice and liver biopsy revealed exclusively NePC. Targeted next generation sequencing (NGS) from formalin-fixed paraffin-embedded (FFPE)-isolated DNA was performed from the diagnostic prostate biopsy and the liver biopsy at progression.

**Intervention:**

Androgen deprivation therapy for adenocarcinoma followed by multiagent chemotherapy for NePC.

**Main outcomes and measures:**

Identification of the mutational landscape in primary adenocarcinoma and NePC liver metastasis. Whether the NePC arose independently or was derived from the primary adenocarcinoma was considered based on mutational profiles.

**Results:**

A deleterious somatic *SMAD4* L535fs variant was present in both prostate and liver specimens; however, a *TP53* R282W mutation was exclusively enriched in the liver specimen. Copy number analysis identified concordant, low-level alterations in both specimens, with focal *MYCL* amplification and homozygous *PTEN*, *RB1*, and *MAP2K4* losses identified exclusively in the NePC specimen. Integration with published genomic profiles identified *MYCL* as a recurrently amplified in NePC.

**Conclusions and relevance:**

NGS of routine biopsy samples from an exceptional non-responder identified *SMAD4* as a driver of the aggressive course and supports derivation of NePC from primary adenocarcinoma (transdifferentiation).

**Electronic supplementary material:**

The online version of this article (doi:10.1186/s13045-015-0204-7) contains supplementary material, which is available to authorized users.

## Introduction

Precision oncology heralds an era in which tumors are biopsied and profiled in the metastatic setting with the goal of identifying therapeutic targets. Although next generation sequencing (NGS) of “N of 1” cases have identified mechanisms of exceptional response to investigational therapies [1–4], such approaches have largely not been applied to exceptional non-responders. Likewise, NGS profiling of pre-/post-treatment samples in cases with marked histologic progression, which enables assessment of progression mechanisms, is challenging due to difficulties in obtaining and assessing routine diagnostic biopsy samples. Here, we describe NGS assessment of routine clinical samples from a patient diagnosed with metastatic PCa at a young age who rapidly progressed and died from disease approximately 1 year from diagnosis. Importantly, while his primary tumor exclusively contained conventional prostatic adenocarcinoma, a post-treatment liver metastasis biopsy exclusively contained prostatic small cell carcinoma/NePC. Hence, this case provided a unique opportunity to assess the utility of NGS-based profiling of serial routine biopsy specimens from an “exceptional non-responder” who showed rapid histologic progression during treatment.

## Methods

### Study oversight

The patient signed a consent form to participate in an IRB-approved research study to sequence tumor and germline DNA from men presenting with metastatic PCa before age 60 years.

### Tumor sequencing and analysis

Post-mortem, we performed targeted next generation sequencing (NGS) on DNA and RNA co-isolated from macrodissected formalin-fixed paraffin-embedded (FFPE) tissue sections from the original diagnostic prostate biopsy specimen (PR-259) and the post-treatment liver biopsy specimen (PR-258). Multiplexed PCR-based NGS (Ampliseq) was performed using 40-ng DNA and the Ion Torrent Comprehensive Cancer Panel (CCP), which targets the coding region of 409 cancer-related genes with 15,992 amplicons (1,688,650 targeted bases) [5, 6]. Multiplexed PCR-based NGS (Ampliseq) was also performed using 20 ng RNA and the RNA component of the Oncomine Comprhensive Panel (OCP), which uses a total of 154 primer pairs to target known gene fusion isoforms, including those involving recurrent 5’ (*TMPRSS2*, *SLC45A3*, *C15ORF21*) and 3’ (*ERG*, *ETV1*, *ETV4*, *ETV5*, and *BRAF*) fusion partners in PCa [7]. Detailed description of sequencing, data analysis using validated pipelines, sequencing statistics, and all identified high-confidence somatic variants are given in the Supplement (Additional file 1 and Tables 1 and 2).

**Table 1:** Sequencing statistics for the diagnostic prostate biopsy sample containing conventional adenocarcinoma (PR-259) and subsequent liver metastasis with small cell/neuroendocrine carcinoma (PR-258)

Parameter	PR259	PR258
DNA sequencing		
Mapped reads (*n*)	25,670,652	2,937,737
On target reads (%)	98.1 %	99.0 %
Total aligned base reads	2,672,758,224	322,308,287
Total base reads on target	2,562,288,371	310,618,776
Average base coverage depth	1,517	184
Uniformity of base coverage	51.2 %	90.9 %
Target base coverage at 20×	89.9 %	94.2 %
Target base coverage at 100×	71.7 %	72.4 %
Target bases with no strand bias	93.5 %	93.3 %
Total called variants^a^	2,556	1,177
Variants passing filtering^b^	5	6
Somatic variants^c^	3	4
Prioritized somatic variants^d^	1	2
RNA sequencing		
Total reads (*n*)	66,564	247,655
Uniquely mapped to genome (*%*)	38 %	79 %
Identified gene fusions (*n*)	0	0

^a^Variants called by automated low stringency variant calling

^b^Variants passing filtering of technical artifacts, poorly supported variants, germline SNPs and synonymous/non-coding variants

^c^Variants confirmed as somatic through exome sequencing of germline DNA

^d^Somatic variants prioritized as likely driving oncogenic or tumor suppressive mutations as described in the eMethods

**Table 2:** High confidence, non-synonymous variants identified in the diagnostic prostate biopsy sample containing conventional adenocarcinoma (Dx [PR-259]) and subsequent liver metastasis with small cell/neuroendocrine carcinoma (NePC [PR-258])

					Dx (PR-259)		NePC (PR-258)			
Location	Gene	Ref	Alt	AA change	Var. allele frequency (FAO/FDP)	Read depth (FDP)	Var. allele frequency (FAO/FDP)	Read depth (FDP)	AV_TX	AV_NUC
chr17:7577094	*TP53*	G	A	p.R282W	1.6 %	755	69 %	217	NM_000546	c.C844T
chr18:48604783	*SMAD4*	C	–	p.L535fs	36 %	227	67 %	196	NM_005359	c.1605delC
chr12:49449077	*KMT2D*	T	C	p.K11E	18 %	22	37 %	180	NM_003482	c.A31G
chr5:7878077	*MTRR*	C	G	p.P141R	15 %	89	55 %	142	NM_002454	c.C422G
chr19:17953318	*JAK3*	C	T	p.R223H	54 %	76	60 %	70	NM_000215	c.G668A
chr6:32166327	*NOTCH4*	T	C	p.T1543A	42 %	608	35 %	231	NM_004557	c.A4627G

High confidence, non-synonymous variants identified in the diagnostic prostate biopsy sample containing conventional adenocarcinoma (Dx [PR-259]) and paired subsequent liver metastasis with small cell/neuroendocrine carcinoma (NePC [PR-258]) are shown. For each variant, the location (hg19), gene, reference (Ref.) and variant (Alt.) alleles, and amino acid (AA) change info is given. The variant (Var.) allele frequency is the flow-corrected variant containing read count (FAO) divided by flow-corrected read depth (FDP). For the KMT2D and TP53 variants in PR-259, the Var allele frequency was calculated using non-flow-corrected read counts (AO/DP) due to those variants not passing filtering in that sample. The reference sequence (Refseq) and nucleotide (Nuc) change used to derive the AA change are also given. Variants identified as SNPs by exome sequencing of germline DNA are indicated in gray. Prioritized somatic variants are bolded

## Case presentation

**Fig. 1:**
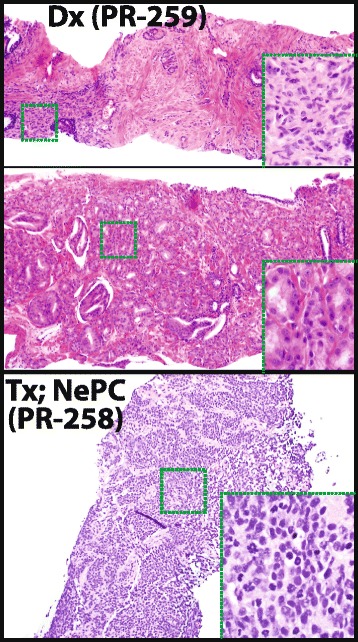
Histology of diagnostic (Dx) prostate biopsy (PR-259) with Gleason score 4 + 5 = 9 adenocarcinoma and subsequent post-treatment (Tx) liver biopsy (PR-258) containing small cell/neuroendocrine prostate carcinoma (NePC). Hematoxylin and eosin-stained diagnostic biopsy cores (*top* and *middle* panels) and liver biopsy (*bottom* panel) are shown. Original magnification 10× (*insets* indicated by *green boxes* 40×)

A 47-year-old male participated in PCa screening due to positive family history of PCa (father). Prostate-specific antigen (PSA) was initially elevated at 13.3 ng/mL, however, prostate biopsy was negative. His PSA rose one year later to 170 ng/mL, and repeat prostate biopsy revealed Gleason 4 + 5 = 9 prostate adenocarcinoma involving all 12 cores (Fig. 1, top and middle panels). Computed tomography (CT) of the abdomen and pelvis showed an enlarged left iliac lymph node but no other metastases. Bone-scan showed metastases involving the lumbar vertebrae. He enrolled in a clinical trial and was treated with an oral anti-androgen along with leuprolide. On this regimen, PSA decreased to a nadir of <4.0 ng/mL over 6 months, and CT scan showed reduction in the size of the enlarged iliac node and normal liver parenchyma.

**Fig. 2:**
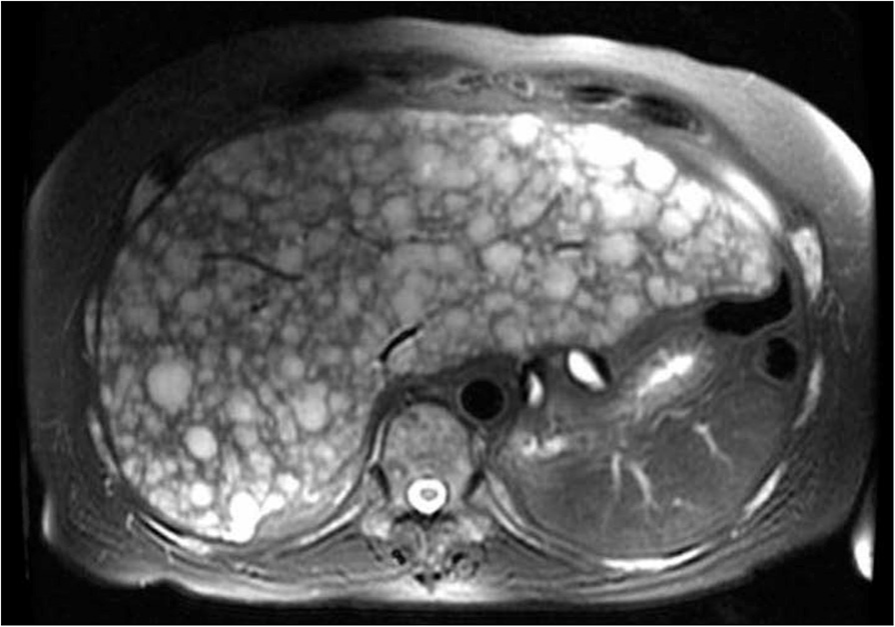
MRI Liver at the time of clinical progression. Axial T2-weighted MRI shows interval development of innumerable solid lesions in the liver, replacing a majority of the parenchyma in both hepatic lobes with development of a pseudocirrhotic appearance of the liver with a nodular surface contour

Two months later, the patient presented with jaundice and was found to have elevated transaminases. MRI showed a pseudocirrhotic appearance of the liver (Fig. 2). Liver biopsy revealed small cell/NePC (Fig. 1, bottom panel). Despite poor performance status and after discussion of risks and benefits, he was initiated on dose-reduced oral etoposide along with carboplatin and continued leuprolide. Although the patient demonstrated an initial clinical response, he elected to end treatment. He died under hospice care 3 months following NePC diagnosis.

## Results

**Fig. 3:**
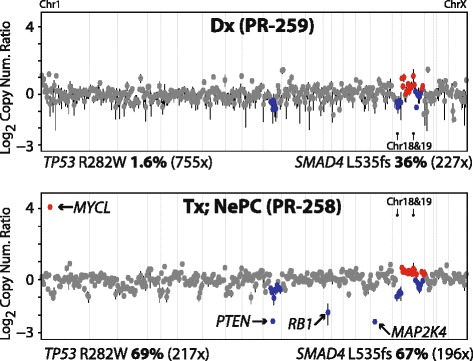
Next generation sequencing (NGS) genomic profiles support transdifferentiaton from prostatic adenocarcinoma (PR-259) to small cell/neuroendocrine prostate carcinoma (NePC, PR-258). Copy number plots and prioritized somatic mutations are shown. *Points* represent the log_2_ copy number ratio for all targeted genes (shown in genome order). Clonal gains and losses are shown in *red* and *blue*, respectively. Prioritized high-level copy number alterations (CNAs) alterations and somatic mutations (with variant allele frequency [%] and coverage depth [*x*]) are indicated. Clonal prioritized *SMAD4* mutation and SCC enriched *TP53* mutation and *MYCL*, *PTEN*, *RB1*, and *MAP2K4* copy number alterations are indicated

NGS of DNA isolated from the routine FFPE diagnostic prostate biopsy specimen (PR-259) and the post-treatment liver biopsy specimen (PR-258) identified a total of two prioritized high-confidence somatic variants. As shown in Fig. 3, a *SMAD4* c1605delC p.L535fs frameshifting variant was present in both PR-259 (36 % variant allele frequency) and PR-258 (67 % variant allele frequency). In contrast, a *TP53* c.C844T p.R282W non-synonymous variant was exclusively called in the NePC specimen (PR-258; 69 % variant allele frequency). This variant was markedly enriched in PR-258, and was only present at a variant allele frequency of 1.6 % (12/755 reads) in the diagnostic pre-treatment specimen (PR-259). These results are consistent with clonal origin and marked enrichment of the *TP53* R282W variant exclusively in the post-treatment NePC specimen. Exome sequencing of germ line DNA isolated from white blood cells confirmed the *TP53* and *SMAD4* variants as somatic (see Table 2).

We next compared NGS-derived copy number profiles between PR-259 and PR-258 using our well-validated approach [5–7]. Copy number profiling revealed broad one copy loss of 10q (containing PTEN), 18q, and a complex alteration on chromosome 19 in both specimens, whereas the post-treatment liver biopsy (PR-258) exclusively demonstrated focal, high-level *MYCL* amplification, and focal homozygous *PTEN*, *RB1*, and *MAP2K4* deletions. Lastly, no gene fusions were identified in either PR-259 or PR-258 from targeted multiplexed PCR-based RNAseq on co-isolated RNA (see Additional file 1). Taken together with the somatic variant analysis, copy number profiling supported the clonal relationship between PR-259 and PR-285, and identified highly enriched, focal, high-level copy number alterations in the post-therapy NePC specimen.

## Discussion

Small cell carcinoma/(NePC) is a rare PCa variant with an aggressive phenotype. Although de novo NePC constitutes <1 % of all PCa, autopsy series of castration-resistant prostate cancer (CRPCa) suggest the presence of NePC in 10–25 % of cases [8]. Despite high initial overall response rates (75–85 %) to platinum combinations, relapse to a chemo-refractory state is nearly universal with a median survival of less than 18 months [9].

Although initially posited as due to clonal selection of malignant neuroendocrine cells [10, 11], recent genetic evidence supports a model of NePC development due to transformation of prostate adenocarcinoma cells to a neuroendocrine phenotype, termed transdifferentiation (see review [12]). Consistent with the concept of a common clonal origin, recurrent prostate adenocarcinoma-specific alterations, such as recurrent ETS gene rearrangements, show concordant status in PCa admixed with NePC, and ETS rearrangement frequency is similar in conventional PCa and NePC [13–15]. Additionally, identical mutations in the DNA-binding domains of *TP53* have been observed in paired prostate adenocarcinoma and NePC [16]. The molecular mechanism of NePC development via transdifferentiation is also supported by a recent report showing gene amplification of *AURKA* and *MYCN* present in 65 % of adenocarcinomas that develop into NePC following ADT whereas only 5 % of unselected adenocarcinomas showing similar amplifications [17]. Lastly, RNAseq profiling in matched NePC and prostate adenocarcinomas showed downregulation of the transcriptional complex REST, which is integral to the repression of neuronal differentiation [18].

Despite the growing evidence in support of transdifferentiation, limited data has been published comparing comprehensive molecular profiling of a primary prostate adenocarcinoma and the subsequent metastatic NePC. Our current study reflects, to our knowledge, the first comprehensive profiling of paired diagnostic FFPE biopsy and subsequent NePC specimens. The presence of a somatic, deleterious *SMAD4* variant in both the diagnostic and NePC specimens is consistent with clonal origin and the transdifferentiation model. Among 816 sequenced PCas in the cBioPortal database [19], six (0.7 %) harbor somatic *SMAD4* mutations, including one sample with two *SMAD4* mutations (Table 3). Importantly, six of the seven reported mutations impact the MH2 domain (including known inactivating and truncating mutations [20,21]) and are recurrent in the COSMIC database [22]. Of note, detailed mechanistic studies demonstrate that *SMAD4* loss leads to an aggressive PCa phenotype in mouse models [23], providing a likely candidate driver of the aggressive phenotype in this exceptional “non-responder.”

**Table 3:** *SMAD4* mutation and *MYCL1* amplification frequency in prostate cancer NGS and copy number profiling studies available in cBioPortal

Study	Sample types	Cases with mutation data (*n*)	Cases with CNA data (*n*)	Cases with *SMAD4* mutations (%)	Cases with *MYCL* amplifications (%)
Prostate (Broad/Cornell 2013)	PCa	57	56	1 (1.8 %)	0 (0 %)
Prostate (TCGA 2015)	PCa	333	492	3 (0.9 %)	0 (0 %)
Prostate (Broad/Cornell 2012)	PCa	112	109	1 (0.9 %)	0 (0 %)
Prostate (MSKCC 2014)	PCa	N/A	104	N/A	0 (0 %)
Prostate (MICH)	PCa and CRPC	61	61	1 (1.6 %)	0 (0 %)
Prostate (MSKCC 2010)	PCa and CRPC	103	194	0 (0 %)	0 (0 %)
Prostate (SU2C)	CRPC	150	150	0 (0 %)	0 (0 %)
Hovelson *et al*. 2015	PCa and CRPC	N/A*	116	N/A	1^ (0.8 %)
Total		816	1282	6 (0.7 %)	1 (0.08 %)

cBioPortal was queried for *SMAD4* mutations and *MYCL* amplifications in prostate cancer tissue profiling studies. Sample types (localized untreated prostate adenocarcinoma [PCa] and/or castration-resistant prostate cancer [CRPC], the number of cases with mutation or copy number alteration (CNA) data, and the number (and %) of cases with SMAD4 mutations and MYCL amplifications are given

^^,^*Data from our recent targeted NGS study (Hovelson et al. 2015) which assessed *MYCL* but not *SMAD4* is also included. The *MYCL* amplified case in that study was small cell/neuroendocrine prostate cancer (NePC). Totals from cBioPortal and our previous study for each parameter is given

The *TP53* p.R282W mutation enrichment and homozygous *RB1* loss in the NePC sample herein supports single gene studies and our recent targeted NGS profiling of eight NePC that show frequent inactivation of these genes in NePC [7, 24, 25]. Likewise, in a recent study, we used qRT-PCR and a combination of exome/targeted NGS to profile distinct conventional PCa and NePC components from an FFPE transurethral resection specimen, which demonstrated enrichment of a *TP53* p.N151fs mutation exclusively in the NePC component [26]. Although both oncogenic and metastasis suppressive roles for *MAP2K4* have been reported in PCa [27–29], its role in NePC has not been described and will require additional investigation.

As described above, recurrent *MYCN* amplifications have been well-described in NePC [17]. Although a recent report identified recurrent *MYCL* amplifications in ~25 % of untreated Gleason score 7 PCa (>2 copies in 8–20 % of malignant glands) [30], clonal, high-level *MYCL* amplifications have not been observed in 1166 prior SNP-, aCGH-, or NGS-based copy number profiled untreated PCa or CRPC in cBioPortal (Table 3). However, in our previous NGS-based profiling of 116 aggressive PCas, we identified a single NePC (of 8 profiled) that harbored a high-level *MCYL* amplification (Additional file 2: Figure S1) [7]. Likewise, copy number profiling of mouse NePC resulting from prostate-specific *p53* and *RB* inactivation identified recurrent *MYCL* gains [31]. *MYCL* amplifications and gene fusions have also been identified and shown to drive proliferation in small cell lung carcinomas [32–34]. Taken together with our previous NGS profiling study, herein we identify recurrent *MYCL1* amplifications in NePC, which will need to be confirmed in additional NePC cohorts.

Alternate mechanisms for the development and maintenance of NE transdifferentiation have been described. The process of “epithelial plasticity” provides evidence for the diverse phenotype of NE-like tumor cells, such as the variable expression of epithelial and NE markers following androgen deprivation [12, 35–37]. This plasticity, which can occur via epithelial-to-mesenchymal transition (EMT) or mesenchymal-to-epithelial transition (MET), is regulated by a complex system of transcriptional networks and signaling pathways. The *TMPRSS2-ERG* fusion gene and certain microRNAs (i.e., miR-200 family) appear to promote the EMT phenotype leading, in part, to the castrate-resistant state [38, 39].

Importantly, neuroendocrine cancers involving other organ sites appear to have distinct molecular aberrations and highlight the need for individualized therapies [40]. For example, telomerase reverse transcriptase (*TERT*) promoter mutations are observed in many human epithelial cancers as well as the vast majority of urothelial neuroendocrine carcinomas, however, they are rarely found in NE-prostate or -lung cancers [41, 42]. Given the molecular heterogeneity of neuroendocrine carcinomas, targeted approaches guided by appropriate biomarker identification, rather than or in addition to cytotoxic therapies, are paramount to improve outcomes [43].

A variety of novel therapeutics targeting receptor tyrosine kinases, mammalian target of rapamycin (mTOR), angiogenesis, cell cycle, epigenetics, and immunotherapy have been tested, largely in small cell lung cancer, with limited success [44, 45]. Specific to the mutational landscape of NEPC, a number of targeted therapies have been investigated in in vitro and in murine models with varied success (see review [46]). Targeting tumor suppressor loss (*TP53*, *RB1*, and *PTEN*) is particularly relevant to NEPC given the high frequency of these alterations. For example, SAR405838, a novel small molecule inhibitor of the oncoprotein murine double minute 2 (MDM2)-*TP53* protein-protein interaction, showed significant activity in wild-type *TP53* murine models, including LNCaP prostate cancer lines [47]. Multiple small molecules that can activate *TP53* are in early phase clinical trials, however, none at this time are recruiting patients with NEPC [46,48]. A phase II study of MLN8237, a small molecule inhibitor of Aurora Kinase A, is currently the only molecularly targeted trial enrolling men with CRPC with neuroendocrine features (NCT01799278).

A limitation of the current report is that it is based on NGS from one patient. Future case studies should consider application of immunohistochemical and morphoproteomic analyses, which might elucidate alternative mechanisms of resistance. The application of these tools has previously revealed means of response and resistance in two cases of refractory Ewing sarcoma that responded to combination therapy with insulin-like growth factor 1 receptor and mTOR inhibition [49].

“N of 1” cases provide unique hypothesis-generating opportunities with the potential to provide new information about pathogenic mechanisms and/or therapeutic response [2, 4]. We suggest that profiling of “exceptional non-responders” and temporally/histologically distinct tumor components [26, 50], as shown herein, may be as informative as “exceptional responder” studies and can exploit the wealth of archived diagnostic tissue specimens. Such studies may be particularly important for identifying the prognostic and predictive associations of rare alterations, such as *SMAD4* mutations in prostate cancer, as well as identifying adaptive alterations associated with treatment resistance/progression such as *MCYL* amplifications.

## Conclusions

Through comprehensive profiling of archived diagnostic and liver biopsy specimens from a single patient with an aggressive clinical course, we identify molecular alterations associated with rapid progression from prostatic adenocarcinoma to NePC, and more broadly identify *MYCL* as a recurrently amplified gene specifically in NePC.

## Additional files

Additional file 1:
**Molecular analysis of transdifferentiation from prostate adenocarcinoma to small cell carcinoma/neuroendocrine prostate cancer.** (DOCX 55 kb) Additional file 2:
**This document provides detailed methodology utilized to conduct the targeted next generation sequencing and corresponding references.** (AI 11975 kb)

## References

[1] Al-Ahmadie H, Iyer G, Hohl M, Asthana S, Inagaki A, Schultz N (2014). Synthetic lethality in ATM-deficient RAD50-mutant tumors underlies outlier response to cancer therapy. Cancer Discov.

[2] Brannon AR, Sawyers CL (2013). "N of 1" case reports in the era of whole-genome sequencing. J Clin Invest.

[3] Iyer G, Hanrahan AJ, Milowsky MI, Al-Ahmadie H, Scott SN, Janakiraman M (2012). Genome sequencing identifies a basis for everolimus sensitivity. Science.

[4] Subbiah IM, Subbiah V (2015). Exceptional responders: in search of the science behind the miracle cancer cures. Future Oncol.

[5] Grasso C, Butler T, Rhodes K, Quist M, Neff TL, Moore S (2015). Assessing copy number alterations in targeted, amplicon-based next-generation sequencing data. J Mol Diagn.

[6] Warrick JI, Hovelson DH, Amin A, Liu CJ, Cani AK, McDaniel AS (2015). Tumor evolution and progression in multifocal and paired non-invasive/invasive urothelial carcinoma. Virchows Arch.

[7] Hovelson DH, McDaniel AS, Cani AK, Johnson B, Rhodes K, Williams PD (2015). Development and validation of a scalable next-generation sequencing system for assessing relevant somatic variants in solid tumors. Neoplasia.

[8] Shah RB, Mehra R, Chinnaiyan AM, Shen R, Ghosh D, Zhou M (2004). Androgen-independent prostate cancer is a heterogeneous group of diseases: lessons from a rapid autopsy program. Cancer Res.

[9] Deorah S, Rao MB, Raman R, Gaitonde K, Donovan JF (2012). Survival of patients with small cell carcinoma of the prostate during 1973–2003: a population-based study. BJU Int.

[10] Yashi M, Terauchi F, Nukui A, Ochi M, Yuzawa M, Hara Y (2006). Small-cell neuroendocrine carcinoma as a variant form of prostate cancer recurrence: a case report and short literature review. Urol Oncol.

[11] Tanaka M, Suzuki Y, Takaoka K, Suzuki N, Murakami S, Matsuzaki O (2001). Progression of prostate cancer to neuroendocrine cell tumor. Int J Urol.

[12] Terry S, Beltran H (2014). The many faces of neuroendocrine differentiation in prostate cancer progression. Front Oncol.

[13] Williamson SR, Zhang S, Yao JL, Huang J, Lopez-Beltran A, Shen S (2011). ERG-TMPRSS2 rearrangement is shared by concurrent prostatic adenocarcinoma and prostatic small cell carcinoma and absent in small cell carcinoma of the urinary bladder: evidence supporting monoclonal origin. Mod Pathol.

[14] Han B, Mehra R, Suleman K, Tomlins SA, Wang L, Singhal N (2009). Characterization of ETS gene aberrations in select histologic variants of prostate carcinoma. Mod Pathol.

[15] Lotan TL, Gupta NS, Wang W, Toubaji A, Haffner MC, Chaux A (2011). ERG gene rearrangements are common in prostatic small cell carcinomas. Mod Pathol.

[16] Hansel DE, Nakayama M, Luo J, Abukhdeir AM, Park BH, Bieberich CJ (2009). Shared TP53 gene mutation in morphologically and phenotypically distinct concurrent primary small cell neuroendocrine carcinoma and adenocarcinoma of the prostate. Prostate.

[17] Beltran H, Rickman DS, Park K, Chae SS, Sboner A, MacDonald TY (2011). Molecular characterization of neuroendocrine prostate cancer and identification of new drug targets. Cancer Discov.

[18] Lapuk AV, Wu C, Wyatt AW, McPherson A, McConeghy BJ, Brahmbhatt S (2012). From sequence to molecular pathology, and a mechanism driving the neuroendocrine phenotype in prostate cancer. J Pathol.

[19] Cerami E, Gao J, Dogrusoz U, Gross BE, Sumer SO, Aksoy BA (2012). The cBio cancer genomics portal: an open platform for exploring multidimensional cancer genomics data. Cancer Discov.

[20] De Bosscher K, Hill CS, Nicolas FJ (2004). Molecular and functional consequences of Smad4 C-terminal missense mutations in colorectal tumour cells. Biochem J.

[21] Maurice D, Pierreux CE, Howell M, Wilentz RE, Owen MJ, Hill CS (2001). Loss of Smad4 function in pancreatic tumors: C-terminal truncation leads to decreased stability. J Biol Chem.

[22] Forbes SA, Beare D, Gunasekaran P, Leung K, Bindal N, Boutselakis H (2015). COSMIC: exploring the world's knowledge of somatic mutations in human cancer. Nucleic Acids Res.

[23] Ding Z, Wu CJ, Chu GC, Xiao Y, Ho D, Zhang J (2011). SMAD4-dependent barrier constrains prostate cancer growth and metastatic progression. Nature.

[24] Tan HL, Sood A, Rahimi HA, Wang W, Gupta N, Hicks J (2014). Rb loss is characteristic of prostatic small cell neuroendocrine carcinoma. Clin Cancer Res.

[25] Chen H, Sun Y, Wu C, Magyar CE, Li X, Cheng L (2012). Pathogenesis of prostatic small cell carcinoma involves the inactivation of the P53 pathway. Endocr Relat Cancer.

[26] Grasso CS, Cani AK, Hovelson DH, Quist MJ, Douville NJ, Yadati V (2015). Integrative molecular profiling of routine clinical prostate cancer specimens. Ann Oncol.

[27] Pavese JM, Ogden IM, Voll EA, Huang X, Xu L, Jovanovic B (2014). Mitogen-activated protein kinase kinase 4 (MAP2K4) promotes human prostate cancer metastasis. PLoS One.

[28] Kim HL, Vander Griend DJ, Yang X, Benson DA, Dubauskas Z, Yoshida BA (2001). Mitogen-activated protein kinase kinase 4 metastasis suppressor gene expression is inversely related to histological pattern in advancing human prostatic cancers. Cancer Res.

[29] Yoshida BA, Dubauskas Z, Chekmareva MA, Christiano TR, Stadler WM, Rinker-Schaeffer CW (1999). Mitogen-activated protein kinase kinase 4/stress-activated protein/Erk kinase 1 (MKK4/SEK1), a prostate cancer metastasis suppressor gene encoded by human chromosome 17. Cancer Res.

[30] Boutros PC, Fraser M, Harding NJ, de Borja R, Trudel D, Lalonde E (2015). Spatial genomic heterogeneity within localized, multifocal prostate cancer. Nat Genet.

[31] Zhou Z, Flesken-Nikitin A, Corney DC, Wang W, Goodrich DW, Roy-Burman P (2006). Synergy of p53 and Rb deficiency in a conditional mouse model for metastatic prostate cancer. Cancer Res.

[32] Peifer M, Fernandez-Cuesta L, Sos ML, George J, Seidel D, Kasper LH (2012). Integrative genome analyses identify key somatic driver mutations of small-cell lung cancer. Nat Genet.

[33] Rudin CM, Durinck S, Stawiski EW, Poirier JT, Modrusan Z, Shames DS (2012). Comprehensive genomic analysis identifies SOX2 as a frequently amplified gene in small-cell lung cancer. Nat Genet.

[34] Varghese AM, Zakowski MF, Yu HA, Won HH, Riely GJ, Krug LM (2014). Small-cell lung cancers in patients who never smoked cigarettes. J Thorac Oncol.

[35] Nieto MA (2013). Epithelial plasticity: a common theme in embryonic and cancer cells. Science.

[36] Nordin A, Wang W, Welen K, Damber JE (2013). Midkine is associated with neuroendocrine differentiation in castration-resistant prostate cancer. Prostate.

[37] Terry S, Maille P, Baaddi H, Kheuang L, Soyeux P, Nicolaiew N (2013). Cross modulation between the androgen receptor axis and protocadherin-PC in mediating neuroendocrine transdifferentiation and therapeutic resistance of prostate cancer. Neoplasia.

[38] Cerasuolo M, Paris D, Iannotti FA, Melck D, Verde R, Mazzarella E (2015). Neuroendocrine transdifferentiation in human prostate cancer cells: an integrated approach. Cancer Res.

[39] Leshem O, Madar S, Kogan-Sakin I, Kamer I, Goldstein I, Brosh R (2011). TMPRSS2/ERG promotes epithelial to mesenchymal transition through the ZEB1/ZEB2 axis in a prostate cancer model. PLoS One.

[40] Zheng X, Liu D, Fallon JT, Zhong M (2015). Distinct genetic alterations in small cell carcinoma from different anatomic sites. Exp Hematol Oncol.

[41] Stoehr R, Taubert H, Zinnall U, Giedl J, Gaisa NT, Burger M (2015). Frequency of TERT promoter mutations in prostate cancer. Pathobiology.

[42] Zheng X, Zhuge J, Bezerra SM, Faraj SF, Munari E, Fallon JT, 3rd, et al. High frequency of TERT promoter mutation in small cell carcinoma of bladder, but not in small cell carcinoma of other origins. J Hematol Oncol. 2014;7:47. 10.1186/s13045-014-0047-7 PMC4223615 25042800

[43] Smith AD, Roda D, Yap TA (2014). Strategies for modern biomarker and drug development in oncology. J Hematol Oncol.

[44] Arcaro A (2015). Targeted therapies for small cell lung cancer: Where do we stand?. Crit Rev Oncol Hematol.

[45] Ehrlich D, Wang B, Lu W, Dowling P, Yuan R (2014). Intratumoral anti-HuD immunotoxin therapy for small cell lung cancer and neuroblastoma. J Hematol Oncol.

[46] Vlachostergios PJ, Papandreou CN (2015). Targeting neuroendocrine prostate cancer: molecular and clinical perspectives. Front Oncol.

[47] Wang S, Sun W, Zhao Y, McEachern D, Meaux I, Barriere C (2014). SAR405838: an optimized inhibitor of MDM2-p53 interaction that induces complete and durable tumor regression. Cancer Res.

[48] Saha MN, Qiu L, Chang H (2013). Targeting p53 by small molecules in hematological malignancies. J Hematol Oncol.

[49] Subbiah V, Naing A, Brown RE, Chen H, Doyle L, LoRusso P (2011). Targeted morphoproteomic profiling of Ewing's sarcoma treated with insulin-like growth factor 1 receptor (IGF1R) inhibitors: response/resistance signatures. PLoS One.

[50] Haffner MC, Mosbruger T, Esopi DM, Fedor H, Heaphy CM, Walker DA (2013). Tracking the clonal origin of lethal prostate cancer. J Clin Invest.

