# Supplementary material for: Comprehensive serial molecular profiling of an “N of 1” exceptional non-responder with metastatic prostate cancer progressing to small cell carcinoma on treatment
Source: J Hematol Oncol. 2015 Oct 6;8:109. doi: 10.1186/s13045-015-0204-7 (PMC4596504; doi:10.1186/s13045-015-0204-7)
Supplement: Additional file 1: — Molecular analysis of transdifferentiation from prostate adenocarcinoma to small cell carcinoma/neuroendocrine prostate cancer. (DOCX 55 kb) [file 13045_2015_204_MOESM1_ESM.docx]

**Molecular Analysis of Transdifferentiation from Prostate Adenocarcinoma to Small Cell Carcinoma/Neuroendocrine Prostate Cancer**

**Supplement**

**eMethods Page 2-3**

**eReferences Page 4**

**eFigure 1 Pages 5**

**eTable 1-3 Pages 6-8**

**eMethods:**

**Targeted Next Generation Sequencing-**

Unstained 10um sections were cut from 4 FFPE diagnostic biopsy blocks (3 sections each) containing conventional prostatic adenocarcinoma (PR-259) and the FFPE liver metastasis biopsy block (8 sections) containing small cell/neuroendocrine prostatic carcinoma (NePC, PR-258). Unstained 10µm sections were carefully manually dissected to enrich for tumor content (final estimated tumor content 60% for both specimens). DNA and RNA were co-isolated from macrodissected tissue using the Qiagen Allprep FFPE DNA/RNA kit (Qiagen, Valencia, CA), as described[^1^](#_ENREF_1)^,^[^2^](#_ENREF_2). Barcoded libraries were generated by multiplexed PCR (Ampliseq, Ion Torrent) from 40ng of DNA per sample using the Comprehensive Cancer Panel (CCP) or 15ng of RNA and sequencing of multiplexed templates was performed using the IonTorrent Proton Sequencer as described[^1^](#_ENREF_1)^,^[^2^](#_ENREF_2).

Data analysis was performed using Torrent Suite (4.2.0) and the Coverage Analysis (or Coverage Analysis RNA) Plug-ins (both v4.0-r73765), along with the Ion Reporter (4.2.0) Fusion analysis workflow essentially and in house validated pipelines essentially as described[^1-6^](#_ENREF_1). For DNA sequencing, alignment was performed using TMAP with default parameters, and variant calling was performed using the Torrent Variant Caller plugin (version 4.2-8-r87740) using default low-stringency somatic variant settings. To prioritize high confidence somatic mutations, we removed the following variants: synonymous or noncoding variants, those with frequencies >0.005 in ESP6500, 1000 Genomes or the Exome Aggregation Consortium (ExAC) database (http://exac.broadinstitute.org), those present in ESP6500, 1000 Genomes or ExAC and variant allele frequencies (FAO/FDP) of 40-60% or >90% (unless occurring at a known hotspot mutation), those with flow corrected read depths (FDP) < 40, flow corrected variant allele containing reads (FAO) < 7, variant allele frequencies (FAO/FDP) < 0.15 in both samples, those occurring at homopolymer runs > 5bp, or extreme skewing of forward/reverse flow-corrected reads supporting the variant allele (FSAF/FSAR <0.2 or >5). Base-level filtering was then applied to candidate somatic variants passing aforementioned criteria to exclude additional technical artifacts, including removal of panel specific errors (called in more than 6% of internally samples sequenced on the CCP unless occurring at a COSMIC recurrent hotspot), variants located at the last mapped base (or outside) of amplicon target regions, variants with the majority of supporting reads harboring excess additional mismatches or indels. Lastly, all variants passing these criteria were then visually inspected in IGV. We have previously shown similar filtering criteria identifies variants passing Sanger sequencing validation with >95% accuracy[^1^](#_ENREF_1)^,^[^4-6^](#_ENREF_4). Variants were assessed in germline DNA subjected to exome sequencing to confirm their status as somatic. Lastly, we prioritized somatic variants in oncogenes occurring at recurrent hotspot residues (>1 report in COSMIC) and deleterious variants (non-sense, frameshift or splice site altering) or hotspot variants in tumor suppressors.

Copy number analysis from total amplicon read counts provided by the Coverage Analysis Plug-in was performed using a validated approach with a pool of individual and composite male genomic FFPE DNA samples as the reference sample for determining copy number ratios as described[^1^](#_ENREF_1)^,^[^3-6^](#_ENREF_3). Genes with log_2_ copy number ratios > 1 or <-1 were considered as high level alterations.

Gene fusion analysis was performed within the Ion Reporter (4.2.0) Fusion analysis workflow, with reads from the RNA AmpliSeq panel aligned using TMAP to a gene reference of targeted chimeric fusion transcripts as well as reference sequences for expression control gene targets as described[^1^](#_ENREF_1).

**eReferences**

1. Hovelson DH, McDaniel AS, Cani AK, et al. Development and validation of a scalable next-generation sequencing system for assessing relevant somatic variants in solid tumors. *Neoplasia.* 2015;17(4):385-399.

2. McDaniel AS, Stall JN, Hovelson DH, et al. Next Generation Sequencing of Tubal Intraepithelial Carcinomas. *JAMA Onc.* In Press.

3. Grasso C, Butler T, Rhodes K, et al. Assessing copy number alterations in targeted, amplicon-based next-generation sequencing data. *J Mol Diagn.* 2015;17(1):53-63.

4. McDaniel AS, Zhai Y, Cho KR, et al. HRAS mutations are frequent in inverted urothelial neoplasms. *Hum Pathol.* 2014;45(9):1957-1965.

5. Warrick JI, Hovelson DH, Amin A, et al. Tumor evolution and progression in multifocal and paired non-invasive/invasive urothelial carcinoma. *Virchows Arch.* 2015;466(3):297-311.

6. Cani AK, Hovelson DH, McDaniel AS, et al. Next-Gen Sequencing Exposes Frequent MED12 Mutations and Actionable Therapeutic Targets in Phyllodes Tumors. *Mol Cancer Res.* 2015;13(4):613-619.
